# Role of Social Support in Improving Infant Feeding Practices in Western Kenya: A Quasi-Experimental Study

**DOI:** 10.9745/GHSP-D-15-00197

**Published:** 2016-03-25

**Authors:** Altrena G Mukuria, Stephanie L Martin, Thaddeus Egondi, Allison Bingham, Faith M Thuita

**Affiliations:** a United States Agency for International Development (USAID) Infant and Young Child Nutrition Project., Washington, DC, USA. Now with USAID Strengthening Partnerships, Results and Innovations in Nutrition Globally (SPRING) Project, Arlington, VA, USA; b USAID Infant and Young Child Nutrition Project, Washington, DC, USA. Now with Cornell University, Division of Nutritional Sciences, Ithaca, NY, USA; c African Population and Health Research Center, Nairobi, Kenya; d PATH, Seattle, WA, USA; e University of Nairobi, School of Public Health, Nairobi, Kenya

## Abstract

**Background:**

We designed and tested an intervention that used dialogue-based groups to engage infants’ fathers and grandmothers to support optimal infant feeding practices. The study’s aim was to test the effectiveness of increased social support by key household influencers on improving mothers’ complementary feeding practices.

**Methods:**

Using a quasi-experimental design, we enrolled mothers, fathers, and grandmothers from households with infants 6–9 months old in 3 rural communities (1 intervention arm with fathers, 1 intervention arm with grandmothers, and 1 comparison arm) in western Kenya. We engaged 79 grandmothers and 85 fathers in separate dialogue groups for 6 months from January to July 2012. They received information on health and nutrition and were encouraged to provide social support to mothers (defined as specific physical actions in the past 2 weeks or material support actions in the past month). We conducted a baseline household survey in December 2011 in the 3 communities and returned to the same households in July 2012 for an endline survey. We used a difference-in-difference (DiD) approach and logistic regression to evaluate the intervention.

**Results:**

We surveyed 554 people at baseline (258 mothers, 165 grandmothers, and 131 fathers) and 509 participants at endline. The percentage of mothers who reported receiving 5 or more social support actions (of a possible 12) ranged from 58% to 66% at baseline in the 3 groups. By endline, the percentage had increased by 25.8 percentage points (*P*=.002) and 32.7 percentage points (*P*=.001) more in the father and the grandmother intervention group, respectively, than in the comparison group. As the number of social support actions increased in the 3 groups, the likelihood of a mother reporting that she had fed her infant the minimum number of meals in the past 24 hours also increased between baseline and endline (odds ratio [OR], 1.14; confidence interval [CI], 1.00 to 1.30; *P*=.047). When taking into account the interaction effects of intervention area and increasing social support over time, we found a significant association in the grandmother intervention area on dietary diversity (OR, 1.19; CI, 1.01 to 1.40; *P*=.04). No significant effects were found on minimum acceptable diet.

**Conclusion:**

Engaging fathers and grandmothers of infants to improve their knowledge of optimal infant feeding practices and to encourage provision of social support to mothers could help improve some feeding practices. Future studies should engage all key household influencers in a family-centered approach to practice and support infant feeding recommendations.

## Abstract (precis)

Fathers and grandmothers who participated in separate nutrition dialogue groups supported mothers to improve infant feeding practices including dietary diversity, food consistency, and use of animal-source foods. Future studies should explore using a family-centered approach that engages mothers together with key household influencers.

## INTRODUCTION

Is family support as important to young child feeding as an individual mother’s caregiving knowledge alone? Many behavior change approaches focus on improving knowledge gaps of mothers and pay little attention to ecological and social factors that may negatively affect a mother’s infant feeding behaviors.^1^ Yet household members, including fathers and grandmothers, exert social influences—sometimes negative because of cultural norms—on a mother’s adoption of optimal infant feeding practices.^2^^–^^5^ Programs that include innovative approaches to engage these key influencers to provide positive social support could be more successful in changing behaviors to improve maternal and child nutrition than programs that focus only on improving mothers’ knowledge.

Household members often exert social influences on a mother’s adoption of optimal infant feeding practices.

Malnutrition is a contributing factor in 45% of under-5 child deaths.^6^ In developing countries, more than 3.5 million children under 5 years of age die each year with undernutrition as an underlying cause. Optimal infant and young child feeding (IYCF) practices contribute greatly to child nutrition. Such optimal practices include exclusive breastfeeding for 6 months and appropriate complementary feeding from 6 months through at least 2 years of age. Some programs have focused on promoting optimal breastfeeding and have shown success, but there has been less programmatic focus on feeding young children from 6 months of age.^7^ Appropriate complementary feeding includes timely initiation of solid/semisolid foods from 6 months of age; increasing the quantity, density, and variety of foods; increasing the frequency of feeding as the child gets older; responsively feeding the child; and ensuring hygienic preparation and feeding of foods. Incorporating animal-source foods in a child’s diet can also help ensure adequate intake of protein, iron, and vitamin A. Appropriate complementary feeding can prevent 6% of all deaths in children 6 to 23 months of age.^8^

In Kenya, high rates of childhood malnutrition began to gradually fall between 2009 and 2014.^9^^,^^10^ Stunting decreased from 35% to 26%, and wasting from 7% to 4%.^10^ Nevertheless, Kenya is one of the 36 countries that carries 90% of the global burden of stunting.^6^ Contributing to Kenya’s high rates of malnutrition are poor IYCF practices. The 2008-09 Demographic and Health Survey (DHS) found that in western Kenya, only 71% of children aged 6 to 23 months were fed the recommended minimum number of times for their age and breastfeeding status, and about 50% of young children were fed adequately diverse diets.^9^ More recent data from the 2014 DHS show that only 21% of children 6 to 23 months old are consuming the minimum acceptable diet in Kenya.^10^ In addition, only 43% of children aged 6 to 35 months consume iron-rich foods.^11^

Kenya is one of the 36 countries that carries 90% of the global burden of stunting.

With high malnutrition rates in Kenya and poor infant feeding practices, community nutrition interventions, and specifically interventions to increase family support for optimal nutrition practices, could help address these problems. The influence of fathers and grandmothers on IYCF practices is well documented in the literature,^2^^,^^4^^,^^12^^,^^13^ and there have been multiple calls to engage these key influencers in maternal and child health (MCH) programs globally^14^ and for IYCF in East Africa.^12^^,^^15^^-^^18^

Gottlieb argued nearly 3 decades ago that social support provides a means to either diffuse information or maintain social norms.^19^ Our formative research found that caregivers, fathers, and grandmothers typically lack up-to-date knowledge of optimal IYCF practices, particularly during the complementary feeding period,^20^^-^^22^ which is consistent with other research in western Kenya.^18^ Other researchers have shown that fathers’ and grandmothers’ lack of support for child feeding is negatively associated with exclusive breastfeeding, diet diversity, meal frequency, and other complementary feeding practices.^17^^,^^23^^,^^24^ There is also evidence that the engagement of grandmothers through group discussion, songs, and stories and of fathers through breastfeeding education and training and men’s group activities can significantly improve IYCF and health behaviors.^25^^-^^28^

**FIGURE 1.**
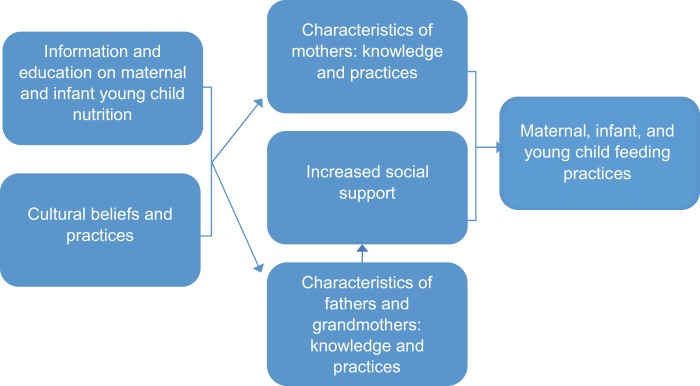
Conceptual Model of Social Support Provided to Mothers Affecting Maternal, Infant, and Young Child Nutrition Practices Cultural beliefs are mitigated by information and education on optimal nutrition practices. Provision of social support along with improved information and education of influencers (i.e., fathers and grandmothers) can impact maternal, infant, and young child feeding practices both indirectly by supporting mothers and directly by the influencers implementing optimal feeding practices themselves.

Employing a socioecological model, we used peer support groups to engage grandmothers and fathers in rural Kenya to support optimal infant feeding practices (Figure 1).^29^^,^^30^ The specific objective of our study was to test the effectiveness of an intervention focused on increasing social support by fathers and grandmothers to improve mothers’ complementary feeding practices in rural communities in western Kenya. The hypothesis guiding this study was that families participating in activities to engage grandmothers or fathers in nutrition would be more likely to adopt better IYCF practices.

We used peer support groups to engage grandmothers and fathers in rural Kenya to support optimal infant feeding practices.

## METHODS

### Study Design

**FIGURE 2.**
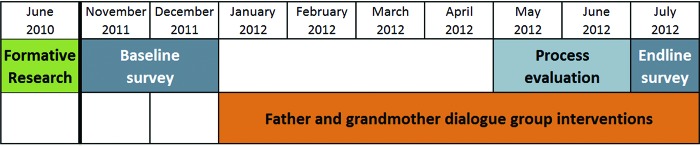
Study Timeline Source: Thuita et al., 2015 ^31^

We conducted a multi-phased, multi-method study between June 2010 and July 2012 (Figure 2), which included the following components:

- Qualitative **formative research** to understand nutrition knowledge and practices, social and cultural relations in the community, health and nutrition-related roles and responsibilities in households, and social support. This formative research informed questionnaire development and intervention design.^20^^-^^23^
- Quantitative **baseline survey** on nutrition knowledge, practices, and social support.
- **Community-based interventions**, including dialogue groups with fathers and grandmothers, community mobilization (2 family bazaars and 5 fathers days at local clinics), and a process evaluation.
- Quantitative **endline survey** on nutrition knowledge and practices and social support.

**Figure:**
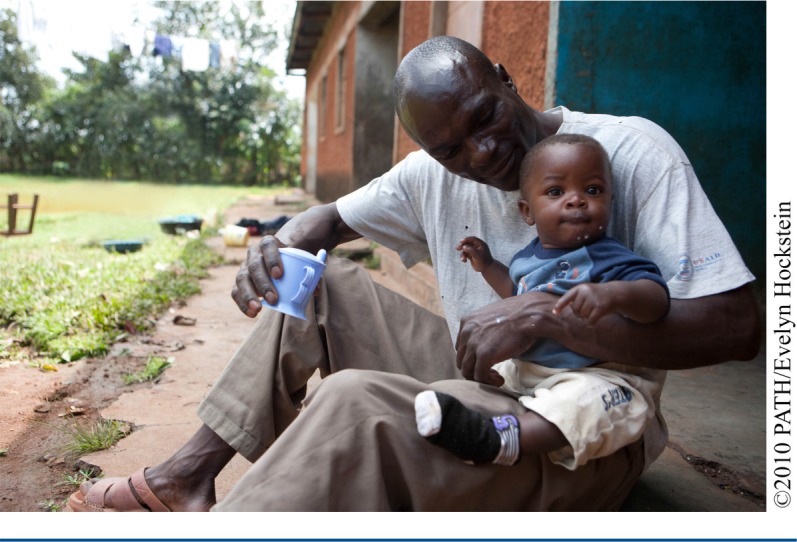
A father in western Kenya feeds his child. Household members such as fathers and grandmothers often exert social influences on a mother’s adoption of optimal infant feeding practices.

This paper reports on the 2 quantitative surveys (baseline and endline), which constituted the impact evaluation. We used a quasi-experimental panel study design with a comparison group that included the survey of the same households. We also used pre- and post-intervention observations of fathers’ and grandmothers’ knowledge of infant feeding practices and provision of social support as well as mothers’ knowledge, practices, and receipt of social support.

### Study Sites

Study sites were selected in 3 sub-locations in 3 districts of Vihiga County in western Kenya. Vihiga County is a densely populated rural area that produces tea and vegetables (commonly, maize and beans).^32^ Interventions were implemented in 2 sub-locations: Kitagwa, Hamisi District, for fathers, and Viguru, Vihiga District, for grandmothers. Findings in these sub-locations were compared with those in Mambai, Emuhaya District, which did not receive any intervention.

The study sites were selected based on discussions with personnel from the Ministry of Health (MOH) and staff from the AIDS, Population and Health Integrated Assistance Plus (APHIA*plus*) Western Kenya Project funded by the United States Agency for International Development (USAID). Selection criteria included the presence of a functional community health unit and APHIA*plus* Western Kenya community-level activities. A functional community health unit is comprised of an employed community health extension worker (CHEW) who works out of a health center or dispensary and supervises a group of volunteer community health workers (CHWs). These CHWs form community health committees and elect their leaders. In functional units, CHWs and committees are actively providing and overseeing community health surveys.

Subsequent baseline survey findings showed that the 3 areas were similar culturally and socially, as well as in regard to residents’ livelihood activities. The comparison area, Mambai, is approximately 35 km from the intervention areas and had a low likelihood of being affected by spillover from the study sites. A full description of the study design and behavior change interventions is provided elsewhere.^31^^,^^33^

### Study Participants

We followed 3 different groups of study participants from the 3 sub-locations:

- Mother/father pairs (intervention group 1 in Kitagwa)
- Mother/grandmother pairs (intervention group 2 in Vigulu)
- Mother/grandmother or mother/father pairs were interviewed but with no intervention (comparison area in Mambai)

We conducted pre- and post-intervention observations of the 3 groups. We selected only households that were willing to participate and that had a child between 6 and 9 months of age. We hypothesized that the 2 intervention groups would report greater social support from the fathers and grandmothers who were engaged, resulting in greater improvements in mothers’ complementary feeding practices, than in the comparison group.

### Ethics Approval

The study was approved by the PATH Research Ethics Committee and the Kenyatta National Hospital/University of Nairobi Ethics and Research Committee. Research assistants reviewed informed consent protocols in the local language or in Kiswahili with the study participants and secured verbal consent before conducting interviews. The research assistants signed a standardized statement that they had followed the protocol for each interview.

### Formative Assessment

Prior to the intervention, the study team conducted a literature review and a qualitative formative assessment to understand the maternal and child nutrition knowledge and practices of mothers, fathers, and grandmothers.^20^^,^^22^ The formative assessment included separate focus group discussions with fathers and grandmothers of children under 2 years of age. Key informant interviews were conducted with community and religious leaders, MOH officers, CHEWs, and women’s group leaders, to explore culturally relevant ways to engage fathers and grandmothers in nutrition interventions.^22^

### Intervention

Using the findings from the formative research, we designed key messages for fathers and grandmothers in households in western Kenya. As per World Health Organization (WHO) and Kenya government infant feeding guidelines,^34^^,^^35^ messages focused on complementary feeding practices, appropriate consistency and variety of foods to be fed to infants (6 to 23 months), age-appropriate meal frequency, and the need for animal-source foods in a child’s diet. Diet diversity and frequent consumption of animal-source foods for pregnant and lactating women were also promoted. We emphasized social support actions that enabled mothers to get adequate rest and seek health services, as well as the provision of foodstuff by fathers and grandmothers.

We used peer dialogue groups to facilitate behavior change among fathers and grandmothers by helping them gain new knowledge, share experiences and reflections, and apply communication and problem-solving skills that in turn would facilitate behavior change in mothers.^36^^,^^37^ We used existing volunteer multi-purpose CHWs operating in the targeted communities and community health units to support the intervention. Each CHW covers 10 to 15 households. To enhance services to the communities, the government encourages development partners to work with CHWs in their sub-location. The CHWs in the 3 study areas conducted a census of households with children 6 to 9 months of age with paternal grandmothers living nearby. Based on the census, we identified and invited mothers, grandmothers, and fathers from a random selection of households in the intervention areas to participate in separate dialogue groups of 8 to 12 participants each. To clearly demonstrate the impact of knowledge and actions of grandmothers and fathers on feeding practices, mothers of the targeted communities were not engaged in dialogue group activities. In sum, 18 dialogue groups were formed, with 79 grandmothers participating in 10 groups in the Viguru sub-location and 85 fathers participating in 8 groups in the Kitagwa sub-location.

The study team designed separate curricula for training fathers and grandmothers, protocols for training CHWs and dialogue group mentors, and dialogue group discussion guides.^38^^-^^41^ Key messages were incorporated into the core intervention package.

Each dialogue group selected one of its members to serve as the group mentor. Mentors were not required to have a certain level of education, but most had at least primary school education. Volunteer CHWs assisted those mentors that were illiterate, for example, to complete monthly reports. Over a 5-day period, mentors were trained in nutrition and health, social support, intrafamilial communication, and gender norms (Box 1). During dialogue group mentor training, trainers modeled adult learning methods, including activities and techniques to encourage participation, experience sharing, and critical reflection by making connections to participant experiences, which mentors then practiced.^31^ Dialogue group mentors were trained in the use of discussion guides and materials and group facilitation techniques.^38^^,^^40^ The mentors received dialogue group facilitation guides with maternal and child nutrition content as well as step-by-step instructions for facilitating activities and probing questions to encourage discussion with their group members.^39^^,^^41^ Before each meeting, mentors selected the discussion topic based on member interest and then facilitated discussions and activities with group members to promote their role supporting recommended nutrition practices, improving relationships and communication with mothers, and for fathers, reflecting on gender norms.^33^ Group members also participated in role plays, problem solving activities, storytelling, and cooking demonstrations; grandmothers also composed songs promoting recommended practices.^32^^,^^33^

**BOX 1. Training Topics**

**Table:** 

Grandmother Dialogue Groups	Father Dialogue Groups
Role of grandmothers in infant and young child feeding and maternal nutrition Overview of maternal and infant and young child nutrition and the local health situation Eating during pregnancy and breastfeeding Early initiation of breastfeeding Exclusive breastfeeding Complementary feeding Preparing food safely Responsive feeding What to do when a child falls ill Mother-to-child transmission of HIV Infant feeding and HIV Supporting mothers with HIV and their families (including referrals and promoting health seeking) Facilitation skills Effective family communication Infant feeding beliefs and myths	Understanding gender Gender roles: behaviors and division of labor and child care in the home Healthy and unhealthy relationships Effective communication Thinking about fatherhood Family care Poor child health “problem tree” What your family eats Understanding maternal and child nutrition Supporting good infant feeding practices during the first 6 months of life Complementary feeding What to do when your child falls ill Mother-to-child transmission of HIV Infant feeding and HIV Disclosure of HIV status (role plays) Men, women, and caregiving Men’s role in health promotion

A volunteer CHW was assigned to each dialogue group to provide support and monitor group activities. Each dialogue group met twice a month for 6 months between January and June 2012. According to members’ preferences, the father groups met in schools, churches, or sometimes homes, whereas the grandmother groups most often met in members’ homes. A small allowance was given to each participant to cater for tea during group meetings (approximately US$1 per meeting, or $2 per month). (The government recommends giving a maximum of $20 per month as an allowance to CHWs engaged in enhanced community health activities.) The participants preferred to receive cash instead of refreshments.

Mentors facilitated dialogue groups using the discussion guides and associated materials.^39^^,^^41^ Members learned about optimal maternal and young child nutrition practices and the role that fathers or grandmothers could play in supporting recommended nutritional practices. They learned and practiced new communication and behavioral skills to support optimal maternal, infant, and young child feeding in their homes and to improve conflict resolution within families.^39^^,^^41^ Members were encouraged to share the information and provide support to mothers in appropriate ways. Dialogue groups fostered discussions in which members shared their experiences and strategies to promote improved nutrition practices in their households.^32^ CHWs supported the dialogue group mentors and closely monitored the groups to assure the quality and accuracy of the information discussed. Each intervention area had 2 paid government CHEWs who oversaw the work of the CHWs and dialogue groups. To provide continual quality control, CHEWs held monthly review meetings with the CHWs and dialogue group mentors.

Grandmothers and fathers participating in dialogue groups learned about optimal maternal and young child nutrition practices and the roles they could play in supporting recommended practices.

Other community mobilization activities were conducted in each study intervention area. These activities included 2 family bazaars (one in each intervention area) where fathers and grandmothers showcased what they were learning through songs, skits, dances, and testimonials. Representatives from the MOH in western Kenya, local leadership, religious leaders, and staff of development partners attended each bazaar along with community members. Dialogue group participants urged other community members to support the mothers to improve nutrition in their households. Mothers gave testimonials of improved familial relationships and increased provision of nutritious foods by their husbands and mothers-in-law. Food demonstration tents promoted dietary diversity coupled with a display of locally available highly nutritious foods for pregnant and lactating mothers and children under 2 years of age.

Five “Fathers Days” were held at local clinics to increase men’s comfort and understanding of maternal and child health services. These Fathers Days were hosted by health facility staff and included health talks by male CHEWs. Fathers were encouraged to accompany their wives and children to the clinic and participate in growth monitoring sessions, and they were given opportunities to ask questions and receive advice from health staff.

There were no dialogue groups in the comparison area. Following the baseline survey, mothers in all 3 locations received 1 home counseling visit from a CHW on maternal and child nutrition. At the end of the visit, the CHWs gave brochures with key messages to each mother in the sample. The mothers were surveyed at baseline and endline to assess any changes in their knowledge and practices that may have been positively influenced by grandmothers or fathers (see below).

### Baseline and Endline Surveys

In December 2011, the study team conducted a household interview survey (baseline) in each of the 3 communities. Using the community census completed by CHWs to identify eligible households, we randomly selected 86 households from each of the 3 communities to achieve a final sample of 69 participants in each group. (Surveyed households were the same households that were included in the intervention and comparison groups.) At endline, we returned to the same households covered at baseline and interviewed those who were available.

Findings from the formative research guided the development of survey tools.^20^^-^^23^ We developed separate tools for interviewing mothers, fathers, and grandmothers. We assessed knowledge and practices of mothers, fathers, and grandmothers in relation to breastfeeding and complementary feeding. In addition, we assessed the quantity of social support provided by grandmothers and fathers in all 3 communities as well as mothers’ perceptions of the social support they received from fathers and grandmothers.

For the endline survey conducted in July 2012, research team members interviewed grandmothers, fathers, and mothers in their homes. These family members were from the same eligible households that were recruited at baseline. They assessed grandmothers’ and fathers’ knowledge and provision of social support as well as mothers’ knowledge, infant feeding practices, and receipt of social support.

### Measurement

#### Social Support Index

Social support is a multidimensional construct. In this study we included 2 domains of social support: receipt of social support (as reported by mothers only) and provision of social support (as reported by grandmothers and fathers).

An overall social support index was generated based on responses to questions about specific social support actions over a specified period of time (Box 2). Grandmothers and fathers were asked in the baseline and endline surveys whether they had provided any support actions to mothers. Mothers were asked about the support actions they had received from the grandmothers and fathers during the past 2 weeks. Based on the formative research and on previous research by the lead author in Nairobi, Kenya,^5^^,^^22^ the index was initially categorized based on the total reported number of social support actions provided to mothers and the number of each of the following types of support:

- **Accompaniment:** In the past 2 weeks, accompanied the mother of the child to a clinic, community event, church meeting, or women’s group meeting (grandmothers only for the latter).
- **Physical support:** In the past 2 weeks, provided help with shopping/marketing, collecting firewood, washing clothes, cooking food or making tea, playing or taking care of child for at least 1 hour, bathing child, or going to the farm.
- **Advice:** In the past 2 weeks, talked with the mother of the child about how the child is being fed or growing.
- **Financial support:** In the past 2 weeks, provided money to buy food.
- **Material support:** In the past month, bought milk, bought or gave meat, or bought or gave fruits for the family (mother and child).

**BOX 2. Social Support Actions**

Based on previous infant feeding research conducted in Kenya,^5^^,^^22^ we included a number of questions in the baseline and endline questionnaires to assess social support provided to mothers of infants by the fathers or grandmothers of the infants. The questions comprised 9 key social support actions conducted for the mother in the 2 weeks preceding the study and 3 material support actions conducted in the past month, as follows.

**Past 2 weeks:**

1. Going to the market
2. Collecting firewood or fetching water
3. Washing clothes
4. Cooking food or making tea
5. Taking care or playing with the child while the mother rested for an hour
6. Bathing child when the mother was busy with other duties or away
7. Going to the farm or supervising farm workers
8. Talking with the mother about how the child is fed or growing
9. Giving the mother money to buy food for the family

**Past month:**

1. Buying milk for child
2. Bringing or giving meat for family
3. Bringing or giving fruits for family

Social support provided to mothers could range from accompanying the mother to a clinic and helping with shopping to providing money to buy food.

For this study, we focused on the material, financial, and physical support actions that were more likely to be provided by both fathers and grandmothers. These actions are more dynamic than the other measures, which showed no variability between groups.

#### Complementary Feeding Practices

In this study, complementary feeding practices were dependent variables. We assessed knowledge and practices of complementary feeding for infants 6 to 9 months of age (baseline) and repeated the assessment for the same households when infants were 9 to 18 months old (endline). The discrepancies in ages (not exactly 6 months after the baseline) were due to the reported age by the mothers and not actual birth dates. We assessed the following practices:

- Consistency of foods regularly consumed
- Adherence to minimum acceptable diet, including feeding frequency and dietary diversity in the past 24 hours
- Consumption of animal-source foods in the past 7 days

#### Socioeconomic Index

We constructed a socioeconomic status (SES) index using information collected on durable asset ownership, household possessions (such as clock, radio, television, farm animals, and mobile phones), access to a sanitation facility, and source of water. Initial descriptive analyses were carried out for all of these variables, assessing means and frequencies to help inform decisions on which variables to include in the analysis. Factor analysis was then used to generate a wealth score, which was divided into 3 categories (lowest, middle, and highest).

### Analytic Methods

#### Sample Size

The dietary diversity indicator was used for sample size computation. At the time of the study design (in 2009), preliminary data on infant feeding practices were gathered from the Kenya Demographic and Health Survey. Among children aged 6 to 23 months in Western Province, an estimated 25% received adequately diverse diets—defined as being fed foods from 4 or more food groups per day.^9^ We hypothesized that the intervention areas would demonstrate an improvement of 30 percentage points in infant feeding practices, whereas the comparison area would demonstrate an improvement of 5 percentage points. Therefore, the expected proportions of young children with adequate dietary diversity within the intervention and comparison groups would be 54.7% and 29.7%, respectively. Using this information and assuming a 5% significance level and 80% power, we calculated that a sample size of 69 individuals per group would be required to detect a 25% difference between the intervention and comparison groups for a total sample of 483 participants. The sample size computation was done using EpiInfo version 3.5.1. We recruited a total of 7 groups, including 3 groups of mothers (1 for each site; total of 207 mothers); 2 groups of fathers (2 sites only; total of 138); and 2 groups of grandmothers (2 sites only; total of 138). The sample size was subsequently adjusted to 86 for each group (total 602) to accommodate an anticipated 20% loss to follow-up.

#### Data Analysis

The baseline analysis included a descriptive analysis of household characteristics and mother/infant characteristics by study site. Indicators for grandmothers, fathers, and mothers/infants were generated and compared across the sites using proportions, mean, or median. Comparisons were made using chi-square tests for categorical variables, and nonparametric tests (Mann-Whitney and Kruskal-Wallis tests) were used where the median was reported as a summary measure.

The impact of the intervention was evaluated using a difference-in-difference (DiD) approach (net difference) based on a community-level analysis. This approach considers the difference between baseline and endline in the comparison group versus the difference between baseline and endline in the intervention groups. The DiD odds ratio (OR) is obtained by assessing the difference between the 2 differences. This approach eliminates the difference that may occur over time in the absence of interventions or differences that may be due to sample selection bias.^42^^,^^43^ The significance of the observed DiD was assessed using logistic regression through interaction of study location (representing intervention group) and time period (i.e., baseline or endline). A logistic regression was used to determine if the quantity of social support actions (as reported by mothers) was associated with the minimum number of meals, minimum dietary diversity, and minimum acceptable diet, after adjusting for the study site. Significance levels were set at *P*<.05.

## RESULTS

### Characteristics of the Study Sample

The total sample size (of intervention and comparison group participants) for the baseline survey conducted in November 2011 was 554, comprising 258 mothers, 165 grandmothers, and 131 fathers. Because 45 participants (8.1%) were lost to follow-up, the endline survey, conducted in July 2012, included only 509 participants (Table 1). Those lost to follow-up were well under the expected 20% figure that was used to calculate sample size estimates.

**TABLE 1:** Sample Sizes at Baseline and Endline, by Type of Participant

Study Areas	No. of Participants							
	Mothers		Grandmothers		Fathers		Total	
	Baseline	Endline	Baseline	Endline	Baseline	Endline	Baseline	Endline
Father intervention area (Kitagwa)	92	70	NA	NA	85	75	177	145
Grandmother intervention area (Viguru)	77	71	79	81	NA	NA	156	152
Comparison area (Mambai)	89	76	86	73	46	63	221	212
**Total**	**258**	**217**	**165**	**154**	**131**	**138**	**554**	**509**
Expected sample (69 participants/group)	207		138		138		483	

Baseline characteristics of the study mothers were generally similar across all 3 locations (Table 2). The 3 groups differed significantly, however, for 3 variables: marital status (100% of the mothers in the father intervention area were married compared with 88% in the grandmother intervention area and 79% in the comparison area), mother’s education (a larger percentage of mothers in the comparison group had completed primary and secondary education than in the intervention groups), and spouse’s education (higher in the comparison group than in the intervention groups). Although these measures are highly correlated to SES, there were no statistically significant differences between the groups based on the more direct measure of SES.

**TABLE 2:** Baseline Characteristics of Study Sample Mothers

	Father Intervention Area (Kitagwa) n = 92	Grandmother Intervention Area (Viguru) n = 77	Comparison Area (Mambai) n = 89	*P* Value
Age of mother, mean, years	27.2	27.5	26.5	.63
Sex of child is male, %	48.9	53.2	53.9	.77
Age of child, %				
6 months	37.0	31.2	29.5	.27
7 months	15.2	18.2	22.7	
8 months	26.1	16.9	15.9	
9 months	21.7	33.8	31.8	
Parity, %				
1	25.0	15.6	33.0	.14
2–3	39.1	40.3	34.8	
4+	35.9	44.2	32.9	
Marital status, %				
Married (ever)	100.0	88.2	78.7	.001
Single/never married	0.0	11.8	21.4	
Mother’s education, %				
Primary completed	38.5	48.1	55.1	.001
Secondary completed	11.0	7.8	21.4	
Mother’s occupation, %				
Subsistence farmer	34.8	46.1	30.6	.32
Homemaker (no outside work)	33.7	30.3	38.8	
Outside work	31.5	23.7	30.6	
Spouse’s age, mean, years	32.9	33.2	33.4	.57
Spouse’s education, %				
Primary completed	31.3	47.1	48.6	.03
Secondary completed	21.7	16.2	27.1	
Spouse’s occupation, %				
Subsistence farmer	18.7	30.9	16.9	.09
Employed	81.3	69.1	83.1	
Socioeconomic status, %				
Lowest quartile	31.5	36.4	32.6	.83
Middle quartile	31.5	31.2	37.1	
Highest quartile	37.0	32.5	30.3	

### Infant Feeding Practices

This section summarizes results related to the consistency and variety of foods fed to infants, the frequency of feeding, and the consumption of animal-source foods.

#### Food Consistency

Although solid foods that are thick in texture can provide a high density of nutrients in a small volume, caregivers often give infants cereals and porridges that are watered down to a level that is of little nutritional value, leading to poor growth.^34^ When baseline study participants were shown 2 pictures of porridge on a spoon (one showing thick consistency and one showing thin) and asked to indicate which picture represented the usual consistency of solid foods given to their infant, about one-third of mothers in each of the 3 study areas indicated they gave foods of the appropriate consistency (Table 3). At endline, improvements were reported in all 3 study areas but more so in the father (68.1%) and grandmother (88.7%) groups than in the comparison group (45.8%). The reported increase between baseline and endline was 21.5 percentage points higher in the father group than in the comparison group (OR, 2.4; *P* = .06) and 44.0 percentage points higher in the grandmother group than in the comparison group (OR, 0.8; *P* = .001) (Table 3). Although the age of the child had increased from baseline to endline, and thus the child was more likely to receive thicker food at endline simply because the child was older, the difference in the increase between the mothers’ responses to adequate food consistency in each of the intervention areas versus the comparison area was substantial and, for the mothers in grandmother group area, statistically significant.

Over time, a higher percentage of mothers reported giving their infants the appropriate food consistency in the father and grandmother intervention areas than in the comparison area.

**TABLE 3:** Changes in Mothers’ Reported Infant Feeding Practices, by Intervention Area: Difference-in-Difference Analysis

	Father Intervention Area (Kitagwa)	Grandmother Intervention Area (Viguru)	Comparison Area (Mambai)	Father vs. Comparison Area			Grandmother vs. Comparison Area		
				DiD	OR	*P* Value	DiD	OR	*P* Value
Adequate consistency of food consumed, No. (%)									
Baseline	92 (32.2)	77 (30.3)	89 (31.4)						
Endline	70 (68.1)	71 (88.7)	76 (45.8)	21.5	2.4	.06	44.0	9.8	.001
Minimum no. of meals provided in past 24 hours, No. (%)									
Baseline	92 (71.7)	77 (77.9)	89 (64.0)						
Endline	70 (70.0)	71 (77.5)	76 (64.5)	-2.2	1.3	.59	-0.9	1.5	.47
Dietary diversity (≥4 food groups), No. (%)									
Baseline	92 (44.6)	77 (48.1)	89 (44.9)						
Endline	70 (85.7)	71 (81.7)	76 (72.4)	13.6	2.3	.11	6.1	1.5	.42
Minimum acceptable diet, No. (%)									
Baseline	92 (39.1)	77 (40.3)	89 (31.5)						
Endline	70 (58.6)	71 (60.6)	76 (46.1)	4.9	1.2	.71	5.7	1.2	.66
Animal-source foods consumed on ≥3 days in past 7 days, No. (%)									
Baseline	92 (9.8)	77 (14.3)	89 (11.2)						
Endline	70 (41.4)	71 (42.3)	76 (11.8)	31.0	6.1	.005	27.4	4.1	.03

Abbreviations: DiD, difference-in-difference; OR, odds ratio.

#### Minimum Acceptable Diet

International standards for optimal infant feeding practices for children 6 to 9 months old include a minimum of 2 meals per day and dietary diversity that includes consumption of at least 4 food groups (out of 7). A minimum acceptable diet dictates that a child under 2 years that is currently breastfed consumes food with the minimum frequency and dietary diversity. In the 24 hours preceding the baseline survey, in all study areas, between 64% and 78% of mothers reported that their infants had received the recommended minimum number of meals, and between 45% and 48% of mothers reported that their infants had been fed at least 4 food groups (Table 3). After the intervention, there was virtually no change from baseline in any of the study groups in the percentage of mothers who reported feeding their child the minimum number of meals; the DiD values were not statistically significant in either of the intervention areas vs. the comparison area (Table 3). Although the percentage of mothers who reported providing their infant the minimum dietary diversity almost doubled between baseline and endline in each of the 3 groups, there was no statistically significant difference in the changes between either of the intervention areas and the comparison area (Table 3). Similarly, the percentage of mothers who reported providing the minimum acceptable diet (breastfeeding plus minimum number of meals and minimum dietary diversity) increased in each of the 3 groups between baseline and endline, but there was no statistical significance in the changes between the grandmother and comparison group and between the father and comparison group (Table 3).

#### Animal-Source Foods

Animal-source foods are a good source of protein, iron, and vitamin A, among other nutrients. Having animal-source foods at least 3 days a week would greatly support young child nutrition. At the start of the study, a very low percentage (10% to 14%) of mothers in any of the 3 groups reported that their children had received animal-source foods on 3 or more days in the 7 days prior to the baseline survey (Table 3). After the intervention, the percentage increased in each of the intervention areas (to 41% in the father group and 42% in the grandmother group) while it remained unchanged in the comparison group. In the father group, the increase from baseline to endline was 31.0 percentage points higher than in the comparison group (OR, 6.1; *P* = .005), and in the grandmother group, the increase was 27.4 percentage points higher than in the comparison group (OR, 4.1; *P* = .03) (Table 3).

Reported provision of animal-source foods increased significantly more in each of the intervention areas than in the comparison area.

### Social Support

Almost all mothers (>95%) reported receiving some social support from grandmothers or fathers in all areas at baseline and endline (Table 4). At endline, the percentage increased to 100% in each intervention area while it dropped slightly in the comparison area (from 97% to 95%). The percentage of mothers who reported receiving 5 or more social support actions (of a possible 12 actions) ranged from 58% to 66% at baseline in the 3 groups. By endline, this percentage had increased in all 3 groups, but it had increased by 25.8 percentage points more in the father intervention group than in the comparison group (OR, 13.6; *P* = .002) and by 32.7 percentage points more in the grandmother intervention group than in the comparison group (OR, 18.4; *P* = .001) (Table 4).

**TABLE 4:** Changes in Reported Social Support, by Intervention Area: Difference-in-Difference Analysis

	Father Intervention Area (Kitagwa)	Grandmother Intervention Area (Viguru)	Comparison Area (Mambai)	Father vs. Comparison Area			Grandmother vs. Comparison Area		
				DiD	OR	*P* Value	DiD	OR	*P* Value
**Mothers’ Reported Receipt of Social Support From Child’s Father or Grandmother**									
No. of support actions received, median									
Baseline	5.0	5.0	5.0						
Endline	10.0	10.0	6.0	4.0	–	–	4.0	–	–
Any social support received, No. (%)									
Baseline	92 (97.8)	77 (94.8)	89 (96.6)						
Endline	70 (100.0)	71 (100.0)	76 (94.7)	4.1	–	–	7.1	–	–
5+ social support actions received, No. (%)									
Baseline	92 (65.2)	77 (58.4)	89 (66.3)						
Endline	70 (97.1)	71 (97.2)	76 (72.4)	25.8	13.6	.002	32.7	18.4	.001
**Fathers’ Reported Provision of Social Support to Child’s Mother**									
Any social support provided by fathers, No. (%)									
Baseline	85 (88.2)	NA	46 (84.8)						
Endline	75 (100.0)	NA	63 (90.5)	6.1	–	–	NA	NA	NA
5+ social support actions provided by fathers, No. (%)									
Baseline	85 (63.5)	NA	46 (54.3)						
Endline	75 (98.7)	NA	63 (52.4)	37.1	46.0	.001	NA	NA	NA
5+ physical support actions provided by fathers, No. (%)									
Baseline	85 (18.8)	NA	46 (21.7)						
Endline	75 (96.0)	NA	63 (44.4)	54.5	35.9	.001	NA	NA	NA
3+ material support actions provided by fathers, No. (%)									
Baseline	85 (38.8)	NA	46 (45.7)						
Endline	75 (96.0)	NA	63 (69.8)	33.0	13.7	.001	NA	NA	NA
**Grandmothers’ Reported Provision of Social Support to Child’s Mother**									
Any social support provided by grandmothers, No. (%)									
Baseline	NA	79 (86.1)	86 (94.2)						
Endline	NA	81 (97.5)	73 (97.3)	NA	NA	NA	8.4	2.9	.36
5+ social support actions provided by grandmothers, No. (%)									
Baseline	NA	79 (60.8)	86 (67.4)						
Endline	NA	81 (90.1)	73 (86.3)	NA	NA	NA	10.4	1.9	.27
5+ physical support actions provided by grandmothers, No. (%)									
Baseline	NA	79 (60.8)	86 (67.4)						
Endline	NA	81 (64.2)	73 (38.4)	NA	NA	NA	36.6	4.4	.002
3+ material support actions provided by grandmothers, No. (%)									
Baseline	NA	79 (10.1)	86 (23.3)						
Endline	NA	81 (72.8)	73 (57.5)	NA	NA	NA	28.4	5.3	.003

Abbreviations: DiD, difference-in-difference; OR, odds ratio.

(–): No values reported either because the indicator is reported in terms of median or because there is 100% response; in either case, logistic regression cannot be fitted.

Mothers’ receipt of 5 or more social support actions increased significantly more over time in the father and grandmother intervention areas than in the comparison area.

In both the comparison area and the grandmother intervention area, almost all grandmothers (≥86%) reported providing some social support to the mothers of the children at baseline (Table 4). By endline, the percentage had increased by 8.4 percentage points more in the grandmother area than in the comparison area, but the difference was not statistically significant (OR, 2.9; *P* = .36). However, in the grandmother intervention area, the percentage of grandmothers reporting provision of 5 or more physical support actions increased from baseline to endline by 36.6 percentage points more than in the comparison area (OR, 4.4; *P* = .002), and the percentage of grandmothers reporting provision of 3 or more material support actions increased by 28.4 percentage points more than in the comparison area (OR, 5.3; *P* = .003) (Table 4).

The intervention seemed to have an even greater impact on fathers’ reported provision of social support. The percentage of fathers reporting provision of 5 or more social support actions in the past week increased between baseline and endline by 37.1 percentage points more in the father group than in the comparison group (OR, 46.0; *P* = .001); provision of 5 or more physical support actions increased by 54.5 percentage points more (OR, 35.9; *P* = .001); and provision of 3 or more material support actions increased by 33.0 percentage points more (OR, 13.7; *P* = .001) (Table 4).

### Association Between Social Support and Infant Feeding Practices

Table 5 reports the logistic regression analysis of the quantity of social support on infant feeding practices. As the number of social support actions increased in the 3 study groups, the likelihood of a mother reporting that she had fed her infant the minimum number of meals in the past 24 hours also increased significantly (OR, 1.14; CI, 1.00 to 1.30; *P* = .047) between baseline and endline. When comparing the grandmother intervention area with the comparison area (without specifically taking into account social support), a mother in the grandmother intervention area was significantly more likely to report having provided the minimum number of meals than a mother in the comparison area (OR, 5.07; CI, 1.56 to 16.50; *P* = .007). The effect in the father intervention area was not statistically significant (OR, 2.94; CI, 0.98 to 8.83; *P* = .055). When taking into account the interaction effects of increasing total social support actions over time and place (i.e., intervention area), there was no effect on the minimum number of meals.

**TABLE 5:** Influence of Reported Social Support Received by Mothers on Infant Feeding Practices

	Min. No. of Meals		Min. Dietary Diversity		Min. Acceptable Diet	
	OR (CI)	*P* Value	OR	*P* Value	OR (CI)	*P* Value
No. of social support actions	1.14 (1.00, 1.30)	.047	1.07 (0.95, 1.21)	.24	1.06 (0.94, 1.20)	.32
Study site; ref: comparison area (Mambai)						
Father intervention area (Kitagwa)	2.94 (0.98, 8.83)	.055	0.4 (0.14, 1.15)	.09	0.95 (0.33, 2.71)	.92
Grandmother intervention area (Viguru)	5.07 (1.56, 16.50)	.007	0.38 (0.13, 1.13)	.08	1.00 (0.35, 2.90)	.99
Support * Father intervention area	0.85 (0.73, 1.00)	.045	1.15 (0.98, 1.34)	.08	1.04 (0.90, 1.21)	.58
Support * Grandmother intervention area	0.82 (0.70, 0.97)	.02	1.19 (1.01, 1.40)	.04	1.06 (0.91, 1.23)	.48

Abbreviation: OR, odds ratio.The first row (number of social support actions) indicates the effect of increasing social support in all 3 study groups on the selected infant feeding practices. The second row (study site) compares the effect on infant feeding practices of the father intervention area vs. the comparison area and the grandmother intervention area vs. the comparison area, without specifically taking into account social support. The last set of rows (support * father intervention area; support * grandmother intervention area) takes into account the interaction effects of both intervention area *and* social support on infant feeding practices.

Dietary diversity was not significantly associated with social support in general or with overall intervention area. However, when taking into account the interaction effects of increasing social support over time and place, there was a significant association in the grandmother intervention area (OR, 1.19; CI, 1.01 to 1.40; *P* = .04) (Table 5). Trends in the father intervention area, when taking into account the interaction effects of time and intervention area, were not statistically significant (OR, 1.15; CI, 0.98 to 1.34; *P* = .08). Results for the association between social support and minimum acceptable diet also were not statistically significant in either of the intervention areas. In this analysis, we did not look separately at the quality of social support (material or physical support), which may have had an impact on behaviors more so than the quantity of social support alone. A larger sample size may have strengthened these findings, and a longer intervention period may have increased the full adoption of these practices with current and subsequent children.

## DISCUSSION

In summary, the results of our study demonstrate that increasing positive social support by key influencers such as fathers and grandmothers of infants improved some, but not all, of the targeted infant feeding practices of mothers. Fathers’ and grandmothers’ provision of material and physical support to mothers increased significantly more in the intervention areas than in the comparison area. The quantity of social support actions that mothers received from these household influencers also increased significantly more in the intervention areas than in the comparison area. The effect of increasing social support appears to be influenced by the quality of the social support (material or physical actions) not just the quantity (number of social support actions) provided to mothers.

Increasing positive social support by key household influencers improved some of the targeted infant feeding practices of mothers.

We found a greater increase between baseline and endline in the percentage of mothers who reported feeding thicker and more diverse foods to their infants in the grandmother intervention area than in the comparison area, and greater improvements in reported feeding of animal-source foods in both the grandmother and father intervention areas. This highlights the difference between what mothers can practice based solely on education and information provided to them versus what they can practice when key influencers are encouraged to provide specific material and physical support actions to the mothers.

If major factors influencing women to adopt recommended IYCF practices are largely related to attitudes of and motivation from family members,^44^^-^^47^ then it behooves us to engage grandmothers and fathers effectively. Although our study examined how engaging fathers and grandmothers separately could influence mothers’ infant feeding practices, designing programs that use a family-centered approach with behavior change interventions for mothers, fathers, *and* grandmothers will likely have a greater impact. Formative research findings should be used to identify contextually appropriate activities and messages. Moving from a woman-centered approach to a family-centered or household-based approach may help to reduce barriers and increase uptake and sustainability of optimal nutrition practices. A previous study from western Kenya found that familial relationships affected how women cared for their children and that infant feeding was part of the social context of the family.^48^ Our formative research also confirmed the importance of fathers and grandmothers for key decision making related to child nutrition and their willingness to be better informed and engaged in nutrition-related activities at home.

Engaging grandmothers and fathers in public health programs is of growing interest to program managers and public health researchers,^2^^,^^12^^,^^49^ yet this approach is not practiced widely, and how to do it effectively is rarely discussed in print. The dialogue approach goes beyond transmitting information to individuals to engaging participants in a discussion group that enables reflection, learning by doing, and resolving conflicts through improved communication techniques.^13^ An intergenerational dialogue approach previously proved effective for improving child feeding practices in Malawi.^50^ In Bolivia and Madagascar, using doable actions proved important for changing behaviors.^51^ Our results suggest that a dialogue process can instigate key social support actions by fathers and grandmothers that are valuable to mothers. As support from fathers and grandmothers increased in our study groups, so did improvements in some infant feeding practices. We believe this is a promising intervention pathway for improving infant and child nutrition.

Our process evaluation data indicated that father and grandmother dialogue groups were well received and contributed to active engagement with mothers, especially by the fathers.^32^ Successful engagement requires careful planning and collaboration with community leaders, local health authorities, and professionals so that communities and households have the institutional support needed to increase their control over their children’s health. The community health unit structure can feasibly provide such institutional support.^33^ Dialogue group mentors reported that the dialogue approach promoted intra-household discussions about child nutrition among grandmothers, fathers, and mothers.^33^ This process enabled these key influencers to give mothers physical and material support in an empathetic way that did not disempower women.^31^^,^^33^ Richards et al. describe this social interaction as “intra-household bargaining,” which they argue is an effective social determinant of health.^52^

In addition to specifying and measuring key social support actions, we specified and measured key infant feeding knowledge and actions that we wanted to change. This included frequency of feeding, diversity of feeding, minimum acceptable diets, and frequency of consuming animal-source foods. Reviews of complementary feeding interventions found that interventions that promoted animal-source foods showed a positive effect on nutritional status.^53^^,^^54^ Although our intervention showed effectiveness in changing some infant feeding practices, it may need to also incorporate other activities where food security is a chronic issues, such as food production activities and support for establishment of kitchen gardens.

### Strengths and Limitations

A clear strength of this study was the level of specificity, which enabled the researchers to more accurately measure the association between reported social support and certain infant feeding practices. However, the intervention time was short (6 months), and additional related topics covered during the dialogue groups may have diluted the impact on key infant feeding practices. The dialogue sessions covered many aspects of optimal infant feeding, maternal dietary practices, and HIV and nutrition over a short period of time. More time was spent on topics of great interest to the participants, such as HIV and nutrition.^31^ It is quite likely that participants varied in their ability to remember details to enable them to encourage mothers to adopt new practices.

Our study has other limitations. First, including grandmothers and fathers in separate intervention arms may have weakened the impact on mothers. If we had a fully family-centered approach and engaged both grandmothers and fathers in the same intervention areas but in separate groups, the impact may have been greater. Findings from our process evaluation, however, suggest that this gender separation was a chief contributor to successful father engagement. Second, when measuring the association between social support and infant feeding practices, the aspects of both quality and quantity need to be examined. Third, the intervention and comparison groups were not completely comparable, and the differences may have diluted the impact of the intervention or confounded measures of the effect size. Due to data limitations, the differences between groups could not be factored into the logistic regressions for evaluating the impact of the intervention. Fourth, although we met the threshold for our sample size criteria, the size of the difference between the intervention and comparison groups was smaller than predicted. If we had a larger sample size, we may have been able to document an even greater impact. We may have been able to enroll a larger number of people in each household and community if we included both grandmothers and fathers in 1 intervention area instead of focusing on 1 group in 2 areas. Finally, the intervention ran for only 6 months. If more time were available, we may have had a greater impact on mothers’ practices of feeding their next child. Infant feeding practices rapidly change within the first year of life. Attempting to affect these practices in a short period may require more intensive education and actions involving the entire family. After the initial intervention, there may be a greater impact on a mother’s next child.

## CONCLUSION

This proof-of-concept study has shown it is possible to move beyond a woman-centered approach to engaging key household influencers by providing education and encouraging “doable” actions to improve infant feeding practices. Engaging fathers and grandmothers who are key influencers of mothers’ infant feeding practices can increase not only the quantity but the quality of support for recommended practices. It is premature, however, to recommend scaling-up this intervention at this time. Future studies should use a family-centered approach that engages all key family members (i.e., mothers, fathers, and grandmothers) in tailored activities to practice and support infant feeding recommendations.

## Notes

**Peer Reviewed**
